# Profound Olfactory Dysfunction in Myasthenia Gravis

**DOI:** 10.1371/journal.pone.0045544

**Published:** 2012-10-17

**Authors:** Fidias E. Leon-Sarmiento, Edgardo A. Bayona, Jaime Bayona-Prieto, Allen Osman, Richard L. Doty

**Affiliations:** 1 Smell and Taste Center, Perelman School of Medicine, University of Pennsylvania, Philadelphia, Pennsylvania, United States of America; 2 Department of Otorhinolaryngology: Head and Neck Surgery, Perelman School of Medicine, University of Pennsylvania, Philadelphia, Pennsylvania, United States of America; 3 Mediciencias Research Group, IPS Ramon and Cajal/Universidad Nacional, Bogota, Colombia; 4 Laboratorio de Neurociencias Clinicas y Funcionales, Neuro.net, Bogota, Colombia; 5 Grupo Cirineo-Neurorehabilitacion, Bucaramanga, Colombia; Neuroscience Campus Amsterdam, VU University, The Netherlands

## Abstract

In this study we demonstrate that myasthenia gravis, an autoimmune disease strongly identified with deficient acetylcholine receptor transmission at the post-synaptic neuromuscular junction, is accompanied by a profound loss of olfactory function. Twenty-seven MG patients, 27 matched healthy controls, and 11 patients with polymiositis, a disease with peripheral neuromuscular symptoms analogous to myasthenia gravis with no known central nervous system involvement, were tested. All were administered the University of Pennsylvania Smell Identification Test (UPSIT) and the Picture Identification Test (PIT), a test analogous in content and form to the UPSIT designed to control for non-olfactory cognitive confounds. The UPSIT scores of the myasthenia gravis patients were markedly lower than those of the age- and sex-matched normal controls [respective means (SDs) = 20.15 (6.40) & 35.67 (4.95); p<0.0001], as well as those of the polymiositis patients who scored slightly below the normal range [33.30 (1.42); p<0.0001]. The latter finding, along with direct monitoring of the inhalation of the patients during testing, implies that the MG-related olfactory deficit is unlikely due to difficulties sniffing, per se. All PIT scores were within or near the normal range, although subtle deficits were apparent in both the MG and PM patients, conceivably reflecting influences of mild cognitive impairment. No relationships between performance on the UPSIT and thymectomy, time since diagnosis, type of treatment regimen, or the presence or absence of serum anti-nicotinic or muscarinic antibodies were apparent. Our findings suggest that MG influences olfactory function to the same degree as observed in a number of neurodegenerative diseases in which central nervous system cholinergic dysfunction has been documented.

## Introduction

Myasthenia gravis (Greek μύúς “muscle”, άσθένεια “weakness”; Latin: gravis “serious”) (MG) has been traditionally viewed as solely a peripheral neuromuscular disease characterized by fluctuating fatigue and muscle weakness [1], [2]. Its primary symptoms arise from damage produced by autoantibodies directed against acetylcholine receptors (AChRs) on the postsynaptic neuromuscular junction [3]–[5]. Anti-AChR antibodies can be detected in serum in about 85% of MG patients, whereas the remaining cases are seronegative. However, about 40% of the latter have detectable antibodies against muscle-specific kinase (MuSK), a receptor kinase required for the formation of cholinergic receptors at the neuromuscular junction [3].

The general notion that MG is strictly a peripheral nervous system disease stems historically from findings that this disorder is not accompanied by gross or otherwise obvious brain pathology [6]. Following the discovery that MG is an autoimmune disorder associated with damage to muscle AChRs [3], this view continued following reports that (a) muscle AChR antibodies do not meaningfully cross the blood brain barrier (BBB) [7], (b) MG patients are seronegative for ganglionic neuronal AChR autoantibodies [8], and (c) muscle AChR antibodies do not bind to major cholinergic neuronal receptor subtypes within the human brain [9]. When behavioral and physiological evidence has been presented in support of MG's involvement in the central nervous system (CNS), lack of replication has been noted in some cases and positive findings have been frequently discounted [10]. For example, while some studies have found MG-related deficits in verbal memory relative to controls, others have not [11]. The higher prevalence of depression and anxiety seen in MG patients relative to controls has been interpreted as psychological responses to a debilitating and incapacitating disease, rather than to disease-specific CNS changes [10]. Sleep disturbances, which have been found in some, but not all, MG studies, have been considered to originate “ in the periphery rather than in the CNS, the result of hypoxia caused by oropharyngeal, intercostal and diaphragmatic muscle weakness which may worsen during sleep, especially during REM sleep” [10].

Despite this perspective, there is support for the concept that MG may influence CNS cholinergic processes. Thus, electroencephalographic studies show abnormalities in MG patients [12], as well as in animals with experimental autoimmune MG [13]. Prolonged latencies and decreased amplitudes in visual and auditory evoked potentials have been consistently reported [14], [15]. Importantly, low levels of MG-related antibodies have been detected in the cerebrospinal fluid (CSF) of MG patients which, in most cases, are proportional to serum antibody levels, suggesting they may cross the BBB from the periphery [16]. Brain nicotinic AChRs, most notably α7 and α3-containing subtypes, have been found to bind antibodies from sera of MG patients [17], and immunization against the ganglionic α3 subunit has been found to produce both muscle and neuronal AChR antibodies [18]. MuSK antibodies known to attack the neuromuscular junction have been recently identified in rat brains where they influence hippocampal oscillatory activity and memory consolidation [19].

We now report that MG is associated with a profound loss of olfactory function. We further demonstrate that this dysfunction, which is analogous to that observed in such disorders as Alzheimer's disease (AD), Down syndrome (DS), Parkinson's disease (PD), and the Parkinson dementia complex of Guam (PDG) [28] , is unlikely due to cognitive confounds or deficits in sniffing ability. Although more research is needed to ascertain whether this deficit is directly due to alterations in the functioning of central cholinergic neurons, our findings are concordant with this concept.

## Methods

### Subjects

Twenty seven MG patients [4 men; 23 women; mean (SD) age = 46.3 (15.4)] were individually matched on age and sex to 27 normal controls. Fourteen of the age matches were exact, 10 were within one year, and 3 were within 2 years. The mean (SD) time since initial diagnosis of was 8.3 (7.7) years. An additional control group was comprised of 11 patients with polymyositis (PM) [4 men; 7 women; mean (SD) age = 39 (9.7)]. Idiopathic PM is a neuromuscular disorder with MG-like motor symptoms largely due to chronic muscle inflammation [20], [21]. The MG patients were volunteers from the regional chapter of the MG Colombian Association of MG or from local practitioners in Colombia, the latter also being the source of the PM patients. The controls were healthy volunteers from the same sources who exhibited no neurological deficits upon neurologic examination.

Diagnosis of MG was clinically confirmed by a neurologist trained in neuromuscular diseases and movement disorders and included a positive Tensilon test and electrodiagnostic recordings consistent with MG. In 11 of the 27 cases, antibody testing was performed and was positive for nicotinic (n = 9) or MuSK (n = 2) serum antibodies. According to the Myasthenia Gravis Foundation of America Clinical Classification System [22], six patients were Class I (any eye muscle weakness, possible ptosis, no other evidence of muscle weakness), 11 were Class II (eye muscle weakness of any severity, mild weakness of other muscles), 7 were Class III (eye muscle weakness of any severity, moderate weakness of other muscles), and 3 were Class IV (eye muscle weakness of any severity, severe weakness of other muscles). Ten patients were being treated with cholinesterase inhibitors alone, two with both cholinesterase inhibitors and glucocorticosteroids, and two with other immunosuppressants in addition to both cholinesterase inhibitors and glucocorticosteroids. Exclusion criteria included a history of alcohol or drug abuse, loss of consciousness greater than 5 minutes, significant cardiovascular disease, major psychiatric illness (e.g. schizophrenia), or the presence of neurological disorders other than MG.

The 11 PM patients met the inclusion and exclusion criteria proposed by Bohan et al [23]. Their medical history included adult-onset weakness and the physical examination demonstrated decreased muscle power, which was generally proximal and symmetric. All had elevated creatine kinase levels, typical inflammatory infiltrates on muscle biopsy, and typical electromyographic changes consisting of “myopathic potentials”, denervation changes, and spontaneous discharges. The myopathic changes involved variation in fiber size and occasional increased connective tissue. None had skin changes or dermatomyositis. Repetitive nerve stimulation was performed in the right ulnar nerve of the dominant hand to rule out possible overlap with MG.

All subjects provided written informed consent after receiving a thorough explanation of the study. The study protocol was reviewed and approved by the ethics committee of the Mediciencias Research Group. The study was conducted according to the principles of the Declaration of Helsinki. No subjects were paid for participation.

### Olfactory Testing

Patients were initially asked whether they suffered from any smell dysfunction. The Spanish version of the UPSIT was then administered, a test that produces test values equivalent to those of the North American English version [24]. This standardized forced-choice test has very high internal consistency and test-retest reliability (rs>0.90) and is strongly correlated with odor threshold measures [25]. The UPSIT is comprised of four booklets, each containing 10 microencapsulated odorants, one odorant per page. A multiple-choice question with four response alternatives per item is located above each odorized strip and the subject must choose the alternative that best represents the perceived smell.

Although the UPSIT is typically self-administered, in this study a trained technician individually administered it to every subject. Each stimulus was released in a standardized manner using a pencil tip and placed under the subject's nose. The response alternatives were read to the subject as they were being presented visually, and the task of the subject was to indicate which of the four response alternatives was most similar to the perceived odor. The experimenter encouraged every subject to strongly sniff each stimulus and visually confirmed that adequate sniffing occurred. To be certain that the test measure was not meaningfully confounded by cognitive problems or the lack of knowledge of the concepts employed in the UPSIT, the PIT, a test analogous to the UPSIT except that pictures rather than odors serve as test stimuli, was administered after the application of the UPSIT [26].

The UPSIT has been found useful for monitoring the efficacy of acetyl cholinesterase inhibitor treatment in patients with AD [27] and is sensitive to the olfactory dysfunction of a number of neurodegenerative diseases known to have marked cholinergic dysfunction, including early stage AD, PD, DS, and PDC. Scores on this test differentiate between sporadic PD and such disorders as supranuclear palsy, essential tremor, multiple system atrophy, and MPTP-induced parkinsonism, disorders with comparatively less cholinergic involvement [28].

### Statistical Analyses

The UPSIT scores of the MG patients and age- and sex-matched controls were compared using Analysis of Variance (ANOVA). The difference between each MG and matched control UPSIT score was then used as the dependent measure in a multiple linear regression analysis with the following measures entered into the model in both forward and backward directions: age, MG disease classification (I–IV), thymectomy operation (Y/N), time since diagnosis in years, taking or not taking medication (Y/N), and PIT score. The test scores of the MG and PM patients were compared using Analysis of Covariance (ANCOVA), with age as a covariate since the ages of these two groups differed slightly.

## Results

**Figure 1:**
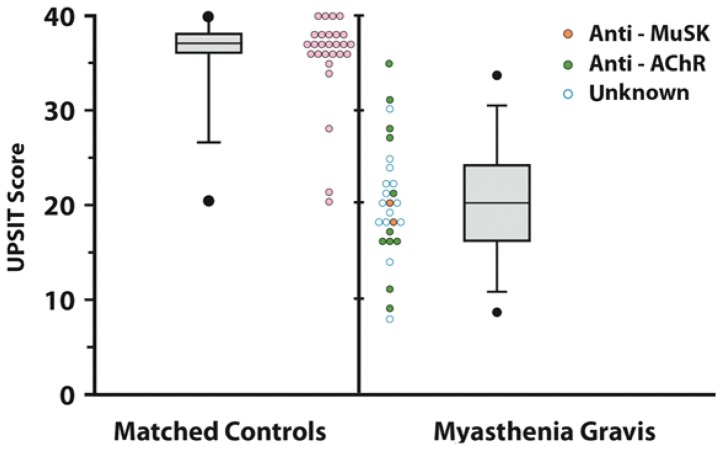
Scores on the University of Pennsylvania Smell Identification Test (UPSIT) for 27 patients with myasthenia gravis and 27 age-, sex-, and race-matched controls. The box plots depict the medians, ranges, the 5th, 25th, 75th, and 95^th^ percentiles of the test scores. The small circles represent individual data points for each subject. Open blue circles indicate patients for whom the results of antibody testing were not available.

The UPSIT scores of the MG patients were much lower than those of their matched normal controls [respective mean (SD) values = 20.15 (6.4) & 35.67 (4.95); F (1,26) = 158.78, p<0.001] (Figure 1), being essentially equivalent to those previously observed for patients with AD, PD, and a number of related disorders [28]. The UPSIT scores of the MG patients were also markedly lower than those of the 11 PM patients, whose UPSIT scores were slightly below those of the controls [respective ANCOVA age-adjusted means (SDs) = 20.62 (4.62) & 33.30 (4.71); group F (1,35) = 56.15, p<0.0001]. No variable included in the multiple linear regression analysis achieved significance at the 0.05 α level, and the UPSIT scores of the MG patients with anti-MuSK and anti-AChR antibodies were distributed throughout the range of the entire group of MG scores (Figure 1).

The PIT scores of the MG group, which were within the normal range, reflected a good understanding of the cognitive elements of the UPSIT [mean (SD) = 37.04 (1.09)]. They were, however, slightly lower than the PIT scores of both the matched controls and the PM patients [respective means (SDs) = 39.48 (0.85) & 38.36 (1.13)], presumably reflecting the subtle cognitive deficits reported by others [29], [30]. Only 15% (i.e., 4/27) of the 27 MG patients reported being aware of olfactory dysfunction before testing. None of the PM patients or the healthy controls complained of smell dysfunction.

## Discussion

This study demonstrates, for the first time, that MG is associated with profound dysfunction of the olfactory system. The dysfunction cannot be explained on the basis of meaningful cognitive confounding, given the high PIT scores of the MG group. Moreover, it is highly unlikely that the results can be explained on the basis of a reduced ability to inhale or sniff, given the near-normal performance on the UPSIT of the PM patients (who would be expected to have similar problems as the MG patients with sniffing) and our careful monitoring of the sniffing behavior of the MG patients – monitoring that has been similarly performed in other studies in which sniffing might be a consideration [31].

It is remarkable that this major sensory deficit has gone unrecognized since MG was defined as a clinical entity in the mid-1800's. This failure likely reflects several factors. First, olfactory testing is rarely performed by neurologists and other physicians and, when performed, is typically cursory, simply having the patient smell one or two odorants. Hence, even when such testing occurs, less-than-total dysfunction can easily go unrecognized. Second, many persons with microsmia are unaware of their dysfunction. In the present study, only 15% of the MG patients were aware of their smell problem before testing, a percent similar to that previously reported in AD (8%) [32], PD (13%) [33], and PDG (13%) [34]. In accord with these earlier studies, those few MG patients who recognized their olfactory deficit had severe dysfunction, i.e., three had UPSIT scores indicative of anosmia (e.g., 11, 16, 16) and one of severe microsmia (e.g., 22).

It should be stressed that many persons with smell loss misidentify their problem as one of taste dysfunction [35]. This reflects the fact that flavor sensations, such as coffee, banana, and chocolate, depend upon retronasal stimulation of the olfactory system during deglutition [36] and have little to do with the taste system, per se. It is noteworthy in this regards that a few case reports have suggested that MG is associated with lessened taste function, although no quantitative testing was performed [37]. The sole empirical study on this topic, which assessed taste thresholds for the bitter-tasting agent phenyl thiocarbamide in 50 MG patients and 50 normal controls, found no evidence of taste loss [38]. This study, along with our test results, suggests that the scattered reports of “taste loss” in MG probably reflect loss of flavor sensations secondary to altered smell function.

The physiological basis of the olfactory dysfunction in MG is not known, although it is important to note that the magnitude of the diminished ability to identify odors found in this study is of the same magnitude as that observed in diseases in which significant CNS cholinergic dysfunction has been demonstrated. This includes AD [32],PD [33], DS [39] and PDG [34]. The nucleus basalis of Meynert (nbM), the major source of acetylcholine (ACh) to the forebrain, is significantly damaged in these disorders [40]–[43]. This contrasts with neurodegenerative diseases with little or no damage to the nbM and with little or no olfactory dysfunction, including progressive supranuclear palsy, amyotrophic lateral sclerosis , and multiple system atrophy [43]–[46].

Under the assumption that cholinergic processes are involved in the decreased smell function of MG, the question arises as to whether such involvement occurs at the periphery, i.e., within the olfactory epithelium, or centrally, i.e., in the olfactory bulb or higher brain structures, or both. Peripheral effects could include cholinergic influences on the amount or composition of the mucus that bathes the cilia of the olfactory receptor cells. Type 3 muscarinic acetylcholine receptors have been found on olfactory receptor cells [47], [48] and non-neuronal microvillar cells express signature markers of choline acetyltransferase (ChAT) and the vesicular acetylcholine transporter. Although ACh released from microvillar cells appears not to enhance olfactory receptor cell activity, it can suppress such activity once it has been initiated by other means [52]. The involvement of more central cholinergic processes receives tangential support from a number of quarters. For example, in a positron emission tomography study of 58 PD patients, Bohnen et al [40] found correlations ranging from 0.55 to 0.63 between UPSIT scores and binding activity of the acetycholinesterase ligand [11 C] methyl-4-piperidinyl propionate within the hippocampus, amygdala, and neocortex. After correction for some misapplied data points, no statistically significant association was observed between UPSIT scores and the dopamine ligand [(11)C]dihydrotetrabenazine vesicular monoamine transporter type 2 in the brain regions that were examined [46]. Additional evidence for associations between olfaction and the central cholinergic system include the discovery of α7-nicotinic AChRs on the nerve terminals of olfactory bulb cells of rodents [49]. Decreased odor detection or discrimination performance is present in mice lacking α7-nicotinic AChRs [50] and in rats receiving infusions of the nicotinic receptor antagonist mecamylamine into their olfactory bulbs [51]. In contrast, increased detection or discrimination performance has been observed in both rats and mice following intraperitoneal injection of acetycholinesterase inhibitors such as physostigmine and neostigmine [52], [53].

Since MG is an autoimmune disorder, it is conceivable that the olfactory deficit involves antibodies directed at cholinergic receptors within various segments of the olfactory pathways. The antibodies could be generated either within or outside of the CNS, or both. As noted in the introduction, the correlation between CSF antibodies and MG serum antibodies has led to suggestions of their origin outside of the CNS. It is of interest that the cross reaction of peripheral antibodies against one or more of the subunits that make up neuronal AChRs can down regulate endothelial AChRs, impairing cholinergic signaling critical for maintaining BBB integrity [54]. Attacks to the α7 subunit increase BBB permeability and, in MG, activate the Ca 2+/calmodulin pathway [55]. P-glycoprotein, which is critical for protecting the BBB [56], is abnormal in the peripheral blood mononuclear cells of MG [57] and could contribute to BBB damage in a manner similar to that documented for PD [58].

In addition to opening up barriers for the penetration of anti-cholinergic antibodies from the periphery into the brain, a compromised BBB is susceptible to penetration of activated T-cells from the periphery [54], [55]. The resultant cascade of inflammatory reactions could potentially have a strong influence on the olfactory system, since there is high metabolic activity within the cells of the olfactory bulb that make them vulnerable to proinflammatory microglia-related pathology induced by abnormal expression of cytokines, enzymes, adhesion molecules, and free radicals [44]. Moreover, CSF and brain interstitial fluids drain into the lymphatic system, including the thymus, along prolongations of the subarachnoid space around the olfactory bulb and other CNS structures [59]. Hence, at least theoretically, T- and B-cells could damage the olfactory pathway as a result of their transit within these fluids, in either a central to peripheral or peripheral to central direction.

Whatever its pathophysiological basis, olfactory dysfunction appears to be a key element of MG. The magnitude of the deficit makes it unlikely that it is an artifact of non-olfactory factors. Our results are in accord with other studies that have suggested that MG has, in fact, CNS correlates [13]–[16], [29], [30], [60]. That being said, studies using much larger samples than that used in the present study may be needed to provide more definitive answers as to whether the dysfunction is influenced by such variables as gender, thymectomy, type of treatment regimen, clinical presentation, or type of anti-AChR antibodies [61], [62]. In addition to the potential for opening up new perspectives on understanding critical elements of MG, the present findings suggest that patients with MG should be counseled regarding their heightened vulnerability to such environmental hazards as spoiled food, leaking natural gas, and fire, as well as the potential nutritional consequences of lessened flavor sensations.
